# Supplementary material for: ER-positive breast cancer cells are poised for RET-mediated endocrine resistance
Source: PLoS One. 2018 Apr 2;13(4):e0194023. doi: 10.1371/journal.pone.0194023 (PMC5880349; doi:10.1371/journal.pone.0194023)
Supplement: S1 Table — PRO-seq was conducted in the indicated cell clone and biological condition. Raw PRO-seq data were sequenced to a read depth >20 million uniquely mapped reads and aligned using established pipelines. (DOCX) [file pone.0194023.s001.docx]

| **Cell Clone** | **Resistance** | **Treatment** | **Time** | **Uniquely mapped reads** |
| --- | --- | --- | --- | --- |
| B7 | TamS | None |  | 21314970 |
| C11 | TamS | None |  | 20333086 |
| G11 | TamR | None |  | 22161480 |
| H9 | TamR | None |  | 23454417 |
| **Total:** |  |  |  | **87,263,953** |

**Supplementary Table 1. PRO-seq data collection and sequencing depth.** PRO-seq was conducted in the indicated cell clone and biological condition. Raw PRO-seq data were sequenced to a read depth >20 million uniquely mapped reads and aligned using established pipelines.
